# HDAC7 promotes the oncogenicity of nasopharyngeal carcinoma cells by miR-4465-EphA2 signaling axis

**DOI:** 10.1038/s41419-020-2521-1

**Published:** 2020-05-06

**Authors:** Qi-Guang Li, Ta Xiao, Wei Zhu, Zheng-Zheng Yu, Xiao-Pu Huang, Hong Yi, Shan-Shan Lu, Yao-Yun Tang, Wei Huang, Zhi-Qiang Xiao

**Affiliations:** 1 Department of Otolaryngology Head and Neck Surgery, Xiangya Hospital, Central South University, Changsha, 410008 China; 2 Research Center of Carcinogenesis and Targeted Therapy, Xiangya Hospital, Central South University, Changsha, 410008 China; 3 The Higher Educational Key Laboratory for Cancer Proteomics and Translational Medicine of Hunan Province, Xiangya Hospital, Central South University, Changsha, 410008 China; 4 Institute of Dermatology, Chinese Academy of Medical Sciences and Peking Union Medical College, Nanjing, 210042 China

**Keywords:** Head and neck cancer, Head and neck cancer

## Abstract

HDAC7 plays a crucial role in cancers, and is the main drug target of several HDAC inhibitors. However, the role and mechanism of HDAC7 in nasopharyngeal carcinoma (NPC) are still unclear. In this study, we observed that HDAC7 was significantly upregulated in the NPC tissues relative to normal nasopharyngeal mucosa (NNM) tissues, HDAC7 expression levels were positively correlated with NPC progression and negatively correlated with patient prognosis, and HDAC7 knockdown dramatically inhibited the in vitro proliferation, migration, and invasion of NPC cells, and the growth of NPC xenografts in mice, indicating the HDAC7 promotes the oncogenicity of NPC. Mechanistically, HDAC7 promoted the in vitro proliferation, migration, and invasion of NPC cells by upregulating EphA2, in which miR-4465 mediated HDAC7-regulating EphA2, a direct target gene of miR-4465. We further showed that miR-4465 was significantly downregulated in the NPC tissues relative to NNM tissues, and inhibited the in vitro proliferation, migration, and invasion of NPC cells by targeting EphA2 expression. Moreover, we observed that the expressions of HDAC7, miR-4465, and EphA2 in NPC tissues were correlated. The results suggest that HDAC7 promotes the oncogenicity of NPC by downregulating miR-4465 and subsequently upregulating EphA2, highlighting HDAC7 as a potential therapeutic target for NPC.

## Introduction

Nasopharyngeal carcinoma (NPC) is a head and neck cancer that shows a distinct endemic distribution with a high prevalence in Southern China and Southeast Asia, and remains one of the leading lethal malignancies in these areas^1^. Radiotherapy is notably effective for control early NPC. However, most of NPC patients are diagnosed at advanced stages due to nonspecific symptoms, in which tumor invasion, and regional and distant metastasis have occurred, and the patients respond poorly to therapy, and their prognosis is dismal^2^. Therefore, it is imperative to explore the underlying mechanism of carcinogenesis and progression of NPC, which could offer novel therapeutic targets.

Histone deacetylases (HDACs) are involved in cancer development and progression. To date, eighteen HDACs have been identified and divided into four classes: class I HDACs (HDAC1–3, 8), class II HDACs (HDAC4–7, 9–10), class III HDACs (Sirtuin1–7), and class IV HDAC (HDAC11)^3,4^. Several HDAC inhibitors (HDACis) have been approved for clinical treatment of hemolymphatic tumors^4–6^. HDACis have also been demonstrated clinical activities either alone or in combination with other agents in solid tumors^5,6^. It is reported that HDACis also possess anti-NPC effects. HDACis, such as Vorinostat, Romidepsin, and MS-275, inhibit NPC cell proliferation, and enhance bortezomib to kill NPC cells^7–9^, and Abexinostat sensitizes NPC cells to cisplatin and radiation^10^. Our previous study reveals that sodium butyrate, a HDAC inhibitor, could induce autophagic death of NPC cells^11^.

HDAC7 regulates cell proliferation, differentiation, apoptosis, migration, and stemness in the physiological and pathological condition^12^. Aberrant expression of HDAC7 has been observed in the lung^13,14^, gastric^15^, breast^16,17^, and ovarian cancer^17^, glioma^18^, and hemolymphatic tumors^19,20^. High HDAC7 expression is correlated with metastasis and poor prognosis^13,15,20^. Furthermore, HDAC7 is the main target of several HDACis, such as Vorinostat^21^, valproic acid^22^, and Trichostatin A (TSA)^23^. However, the role and underlying mechanism of HDAC7 in NPC have not been explored.

Eph receptors are key regulators of both normal development and disease^24^. Perturbation of Eph receptor and its ligand system has been observed in the various human cancers. EphA2 is the most frequently affected Eph receptor in human cancers^25^, and is overexpressed in many human cancers, where it promotes tumor growth, metastasis, and cancer stemness^26–31^. Approaches for targeting downregulation of EphA2 have attracted considerable interest as potential anticancer strategies^31^. The recent study revealed that upregulation of EphA2 by HDAC2 and HDAC4 plays a crucial role in breast cancer, and HDACs-EphA2 signaling axis is a promising therapeutic target for advanced breast cancer^32^. We also found that EphA2 promotes NPC cell invasion, metastasis, and stem properties^33^. But whether HDAC7 regulates EphA2 expression in cancer is unclear.

In the present study, we try to determine whether and how HDAC7 promotes the oncogenicity of NPC. We found that HDAC7 was upregulated in NPC cell lines and tissues, and increases the oncogenicity of NPC cells in vitro and in vivo. Mechanistically, HDAC7 enhanced the oncogenicity of NPC cells by downregulating miR-4465 and subsequently upregulating its target gene EphA2. Our findings suggest that HDAC7 can serve as a potential therapeutic target in NPC.

## Results

### HDAC7 expression is increased in the NPC tissues and correlates with NPC progression and prognosis

**Fig. 1:**
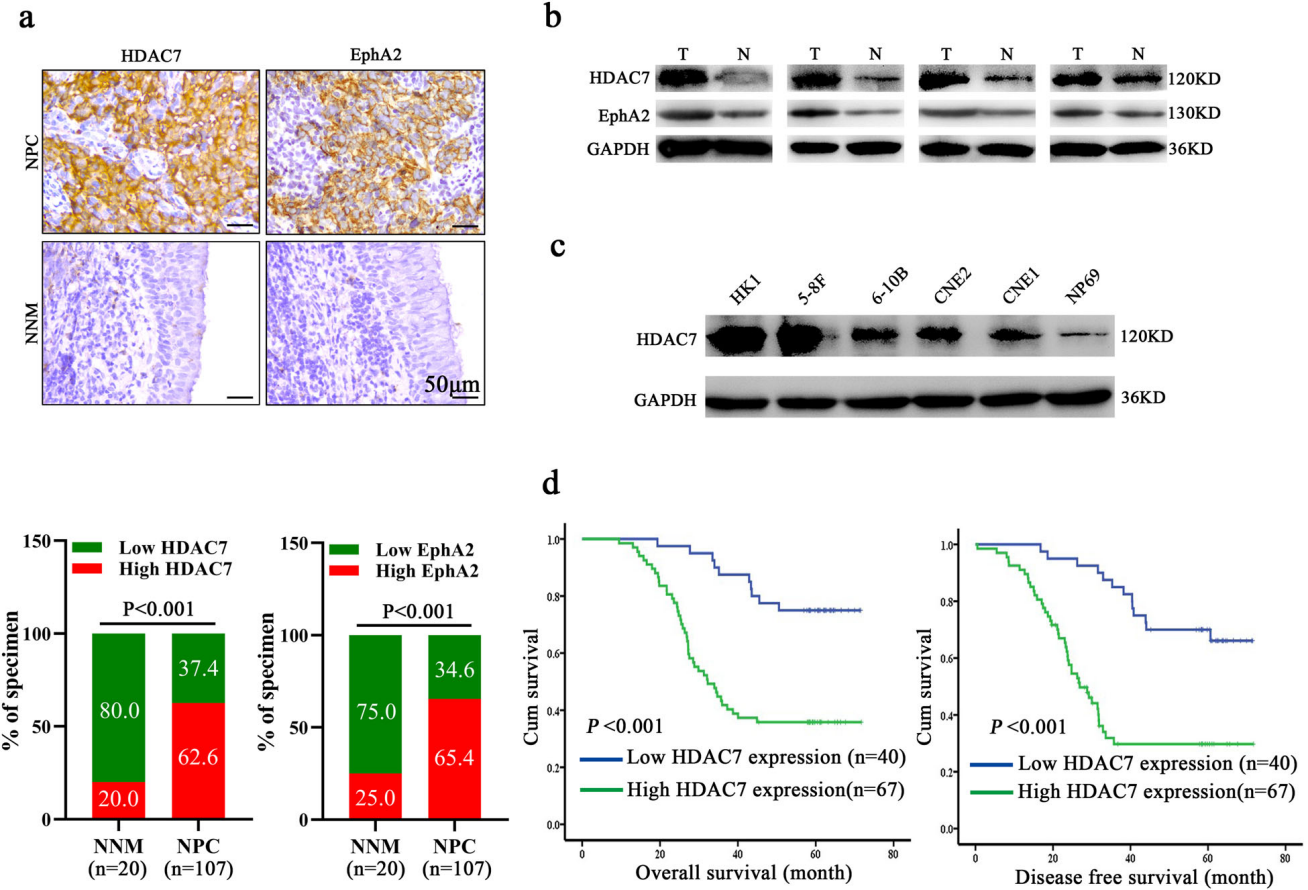
HDAC7 overexpression is correlated with NPC progression and poor patient prognosis. **a** IHC showing the levels of HDAC7 and EphA2 in the NPCs and normal nasopharyngeal mucosa (NNM). Representative IHC images are shown on the top, and statistical analysis is presented on the bottom (*p* < 0.001, Chi-squared test). **b** Western blot showing the levels of HDAC7 and EphA2 in the additional four paired fresh biopsies of NPC and NNM. **c** Western blot showing the levels of HDAC7 in the five NPC cell lines and immortalized normal human nasopharynx epithelial cell line NP69. **d** Kaplan–Meier survival analysis for 107 NPC patients according to HDAC7 expression levels. NPC patients with high HDAC7 expression have a significantly worse overall survival and disease-free survival than those with low HDAC7 expression. The log-rank test was used to calculate *P* value.

Immunohistochemistry (IHC) was used to detect HDAC7 expression in the 107 NPCs and 20 normal nasopharyngeal mucosa (NNM). The results showed that HDAC7 expression was significantly higher in the NPCs relative to NNMs (Fig. 1a). Western blot showed that HDAC7 expression was obviously higher in the additional four fresh NPCs than that in the paired fresh NNMs (Fig. 1b), and in the five NPC cell lines than that in the immortalized normal human nasopharynx epithelial cell line NP69 (Fig. 1c). Subsequently, we analyzed the correlation of HDAC7 expression in the NPC specimens with clinicopathological features and patient prognosis. As shown in Supplementary Table S1, HDAC7 expression was positively correlated with lymphonode and distant metastasis and clinical TNM stage. The Kaplan–Meier plot and Cox proportional hazards regression analysis showed that HDAC7 upregulation was an unfavorable and independent factor for patient overall survival (OS) and disease-free survival (DFS) (Fig. 1d, Supplementary Table S2). Collectively, our data indicate that HDAC7 plays a crucial role in NPC.

### HDAC7 promotes NPC cell proliferation, migration, and invasion in vitro and growth in vivo

**Fig. 2:**
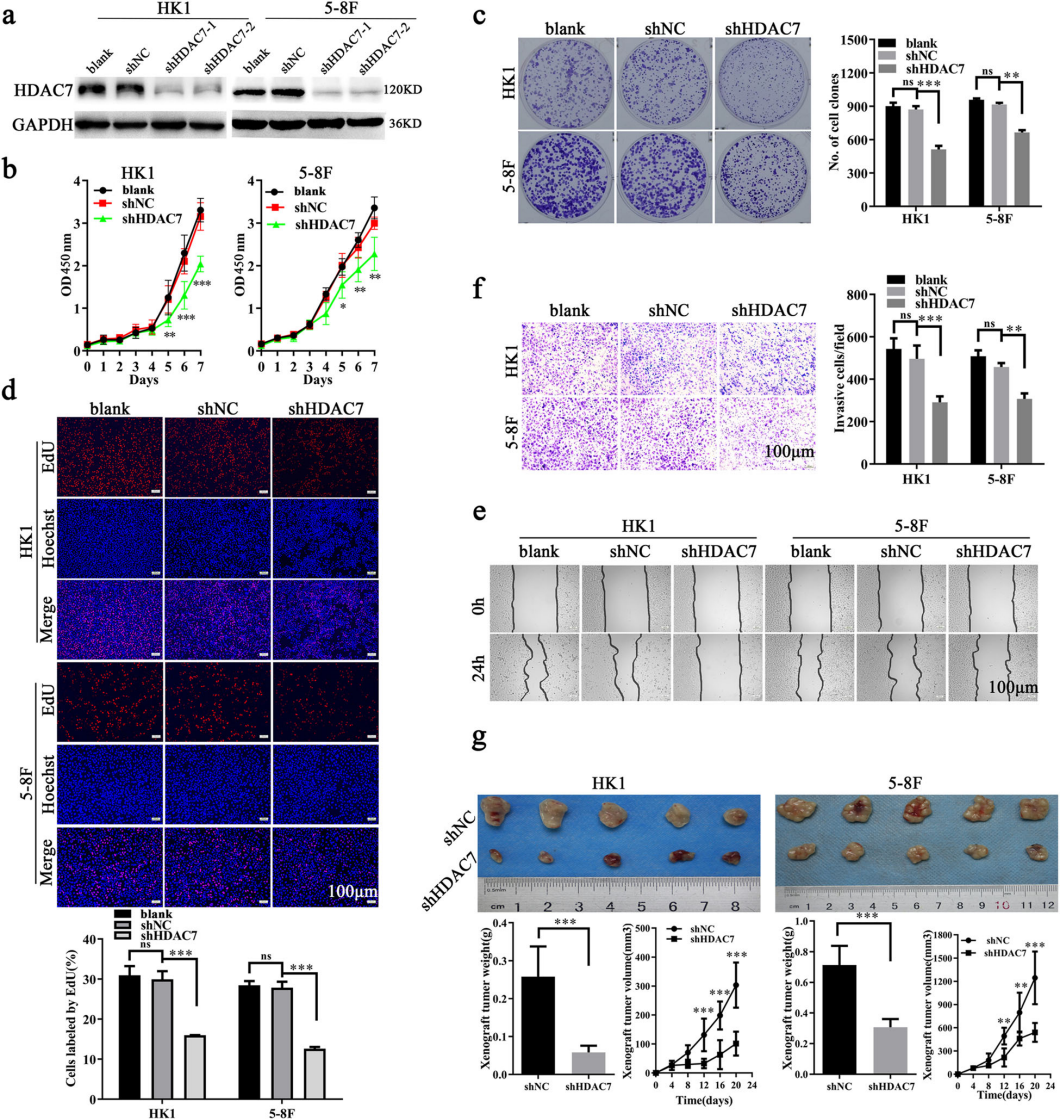
HDAC7 promotes NPC cell proliferation, migration, and invasion in vitro and growth in vitro. **a** Establishment of HK1 and 5–8F cell lines with stable knockdown of HDAC7 by shRNA (shHDAC7) and their control cell lines (shNC). **b–f** HDAC7 knockdown inhibits NPC cell proliferation, migration and invasion in vitro. CCK-8 (**b**), plate clone formation (**c**), and EdU incorporation (**d**) assay showing the proliferation of HK1 and 5–8F cells with shHDAC7 and their control cells. **e** Scratch wound healing showing the migration of HK1 and 5–8F cells with shHDAC7 and their control cells. **f** Transwell Matrigel invasion assay showing the invasion of HK1 and 5–8F cells with shHDAC7 and their control cells. **g** Xenograft growth of HK1 and 5–8F cells with shHDAC7 and their control cells. (Top) The photography of xenograft tumors after 20 days subcutaneous implantation of the cells; (bottom) growth and weight of the xenograft tumors. *n* = 5 mice per group. Means, SDs, and statistical significance are denoted; *P < 0.05; **P < 0.01; ***P< 0.001; ns no significance.

To explore the functions of HDAC7 in NPC, we first established HK1 and 5–8F NPC cell lines with stable knockdown of HDAC7 (HK1 shHDAC7 and 5–8F shHDAC7) by HDAC7 shRNA because both cell lines had high HDAC7 expression (Figs. 1c, 2a), and analyzed the effects of HDAC7 knockdown on NPC cell proliferation, migration, and invasion. CCK-8, plate colony formation, and EdU incorporation labeling assay showed that HDAC7 knockdown significantly decreased NPC cell proliferation (Fig. 2b–d). Scratch wound healing and transwell Matrigel invasion assay showed that HDAC7 knockdown significantly decreased NPC cell migration and invasion in vitro (Fig. 2e, f). Moreover, we transfected HDAC7 expression plasmid into the NPC cells with the knockdown of HDAC7 by siRNA targeting 3′UTR of HDAC7, and observed that reexpression of HDAC7 rescued cell proliferation, migration, and invasion in the NPC cells with HDAC7 knockdown (Supplementary Fig. S1). Collectively, these results demonstrate that HDAC7 promotes NPC cell proliferation, migration, and invasion in vitro.

Subcutaneous tumor formation experiment was performed to detect the effects of HDAC7 knockdown on the in vivo growth of HK1 and 5–8F NPC cells. The results showed that HDAC7 knockdown significantly decreased the growth of NPC cells in nude mice as demonstrated by growth and weight of xenografts (Fig. 2g). The result demonstrates that HDAC7 increases the in vitro growth of NPC cells.

### HDAC7 upregulates EphA2 by suppressing miR-4465 expression in NPC cells

**Fig. 3:**
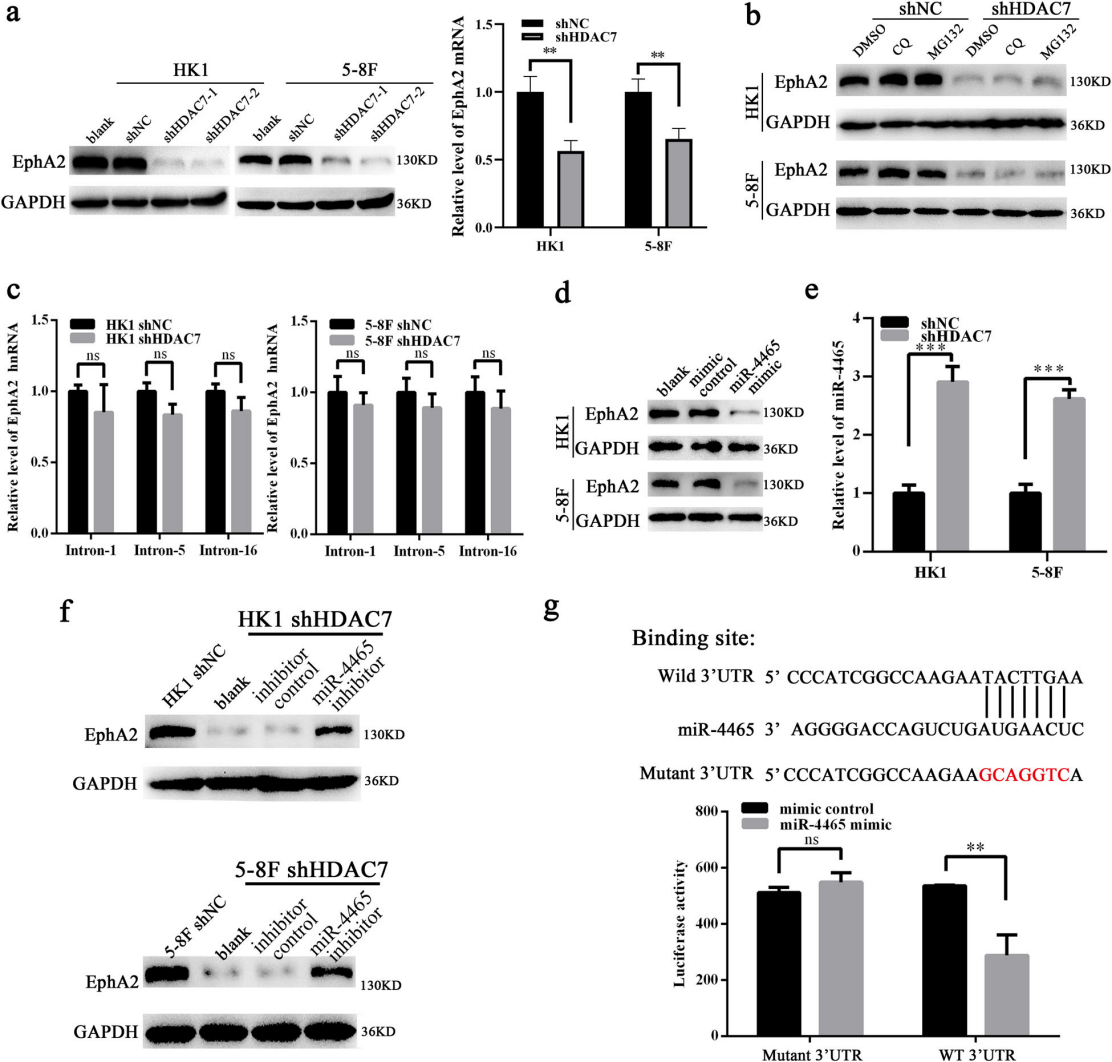
HDAC7 upregulates EphA2 by suppressing miR-4465 expression in NPC cells. **a** Western blot and qRT-PCR showing the levels of EphA2 protein and mRNA in the shHDAC7 HK1 and 5–8F cells and their control cells. **b** Western blot showing the levels of EphA2 in the shHDAC7 HK1 and 5–8F cells and control cells treated by 50 μM chloroquine (CQ) and 20 μM MG132, respectively. **c** QRT-PCR showing the levels of EphA2 hnRNAs in the shHDAC7 HK1 and 5–8F cells and control cells. **d** Western blot showing the levels of EphA2 in the HK1 and 5–8F cells transfected with 100 nM miR-4465 mimic or mimic control. **e** QRT-PCR showing the levels of miR-4465 in the shHDAC7 HK1 and 5–8F cells and control cells. **f** Western blot showing the levels of EphA2 in the shHDAC7 HK1 and 5–8F cells transfected with 200 nM miR-4465 inhibitor or inhibitor control. **g** 3′UTR dual-luciferase reporter assay. (Top) The predicted miR-4465 binding sites in the 3′UTR of wild-type (WT) EphA2 and mutant EphA2 3′UTR are shown; (bottom) luciferase activity of WT and mutant EphA2 3′UTR dual-luciferase reporter vector in the HEK293 cells transfected with control or miR-4465 mimic. Means, SDs, and statistical significance are denoted; **P < 0.01; ***P < 0.001; ns no significance.

The recent study revealed that HDAC2 and HDAC4 promote the oncogenicity of breast cancer cells by upregulating EphA2 expression and phosphorylation^32^. We also found that EphA2 expression and phosphorylation at Ser897 is important for NPC cell invasion, metastasis, and stem properties^33^. Therefore, we detected whether HDAC7 regulates EphA2 expression in the NPC cells. Surprisingly, knockdown of HDAC7 by shRNA remarkably downregulated EphA2 at mRNA and protein levels in the NPC cells (Fig. 3a), implying that decrease of EphA2 protein by HDAC7 knockdown may be attributed to its mRNA downregulation. Moreover, EphA2 protein levels in the NPC cells with HDAC7 knockdown were not restored by either lysosome inhibitor chloroquine (CQ) or proteasome inhibitor MG132 (Fig. 3b), indicating that downregulation of EphA2 by HDAC7 knockdown was not caused by impaired protein stability. These results suggest that HDAC7 upregulates EphA2 by transcriptional or posttranscriptional regulation in the NPC.

Transcriptional and posttranscriptional regulation could be distinguished by detecting the levels of hnRNAs (heterogeneous nuclear RNA), the primary transcripts containing intron sequences, which directly reflect the transcriptional activity of the genes^34^. Therefore, we detected the level of EphA2 hnRNA by qPCR using three pairs of specific primers amplifying intron-1, -5, and -16 of EphA2 respectively. The result showed that no significant expression differences of EphA2 hnRNA were observed between HDAC7 knockdown and control NPC cells (Fig. 3c). The result indicate that HDAC7 regulates EphA2 expression by posttranscriptional mechanism in the NPC cells.

In the posttranscriptional regulation, miRNAs serve as a core regulator to regulation gene expression^35^. Therefore we reasonably speculate that miRNAs mediate HDAC7-regulating EphA2 levels in the NPC cells. For this reason, the potential miRNAs, which could target EphA2, were predicted by using Starbase 2.0, a database for evaluating miRNA-target interaction based on large-scale CLIP-sequence data^36^. Three candidate miRNAs (miR-4465, miR-1297, and miR-26b-5p) with the highest predicted score were selected for experimental validation. The result showed that miR-4465 and miR-26b-5p mimic but not miR-1297 mimic remarkably reduced EphA2 protein levels in NPC cells (Fig. 3d, and Supplementary Fig. S2).

Next, we analyzed the effect of HDAC7 on the expression of miR-4465 and miR-26b-5p in the NPC cells, and observed that HDAC7 knockdown significantly upregulated miR-4465 but not miR-26b-5p in the NPC cells (Fig. 3e, and Supplementary Fig. S3). The upregulation of miR-4465 was also observed in xenografts generated from NPC cells with HDAC7 knockdown (Supplementary Fig. S4). Accordingly, miR-4465 inhibitor but not miR-26b-5p inhibitor could restore the levels of EphA2 protein in NPC cells with HDAC7 knockdown (Fig. 3f, Supplementary Fig. S5). Moreover, we co-transfected a dual-luciferase reporter plasmid with wild-type EphA2 or mutant EphA2 into HEK293 cells with control or miR-4465 mimic. The results revealed a significant reduction in luciferase activity in miR-4465 mimic-transfected cells compared with control mimic-transfected cells, whereas miR-4465 mimic had no obvious effects on the luciferase activity of a dual-luciferase reporter plasmid with mutant EphA2 in the miR-4465 binding site (Fig. 3g), confirming that EphA2 is a direct target of miR-4465. Collectively, our results reveal that HDAC7 upregulates EphA2 via suppressing miR-4465 expression in the NPC cells.

### MiR-4465 functions as a tumor suppressor in NPC

**Fig. 4:**
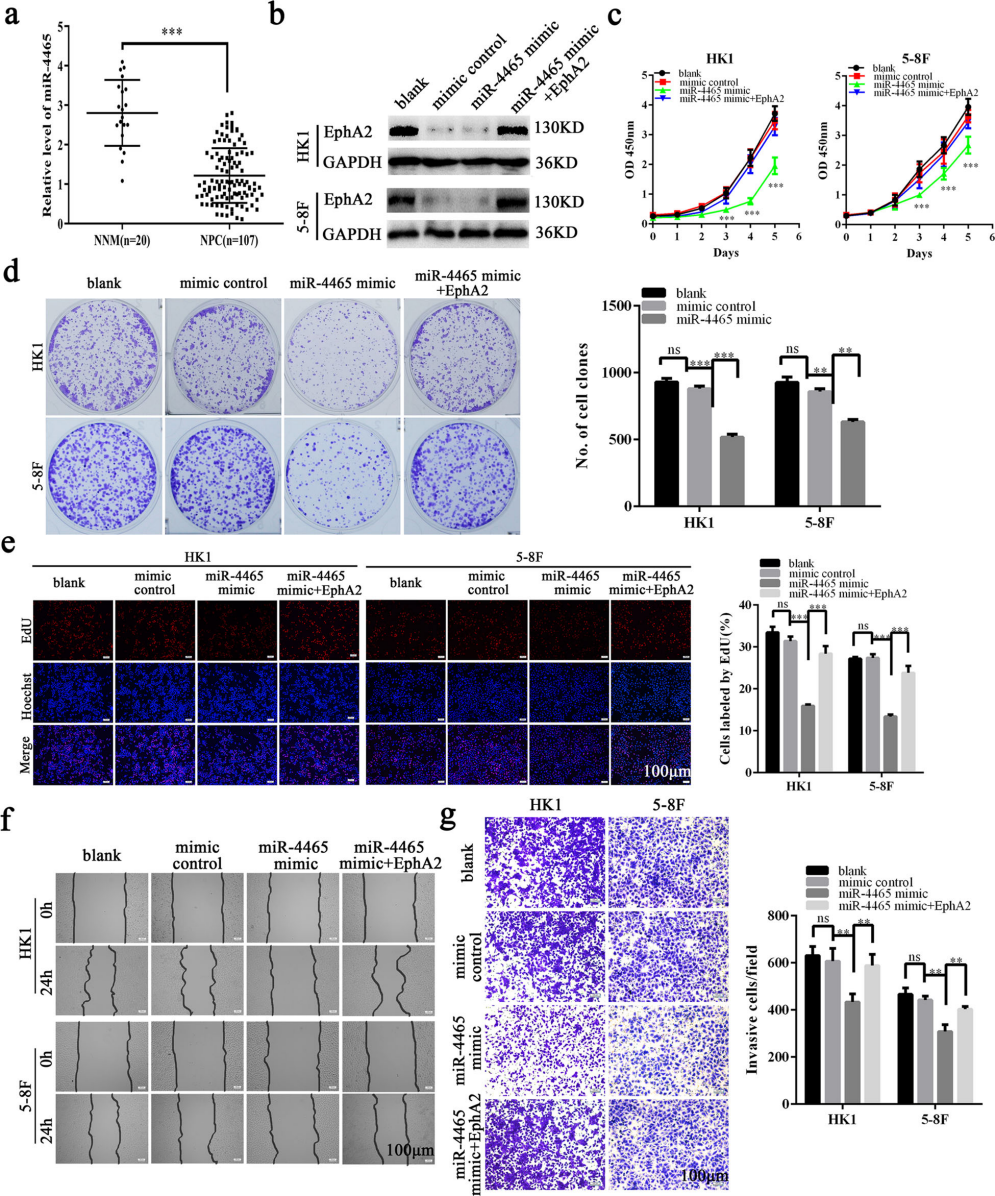
MiR-4465 is downregulated in NPC tissues and inhibits NPC cell proliferation, migration, and invasion in vitro. **a** QRT-PCR analysis of the expression levels of miR-4465 in the 107 NPC tissues and 20 NNM. **b** Western blot showing the levels of EphA2 in the HK1 and 5–8F cells transfected with miR-4465 mimic or co-transfected with miR-4465 mimic and EphA2 expression plasmid, and their control cells. CCK-8 (**c**), plate clone formation (**d**), and EdU incorporation (**e**) assay showing the proliferation of HK1 and 5–8F cells transfected with 100 nM miR-4465 mimic or co-transfected with 100 nM miR-4465 mimic and 1μg/ml EphA2 expression plasmid, and their control cells. Scratch wound healing (**f**) and Transwell Matrigel invasion assay (**g**) showing the migration and invasion of HK1 and 5–8F cells 100 nM miR-4465 mimic or co-transfected with 100 nM miR-4465 mimic and 1 μg/ml EphA2 expression plasmid, and their control cells. Means, SDs, and statistical significance are denoted; **P < 0.01; ***P < 0.001; ns no significance.

Although the previous studies indicate that miR-4465 functions as a tumor suppressor in the ovarian clear cell carcinoma and non-small cell lung cancer^37,38^, its function is unclear in NPC. Therefore, we detected miR-4465 expression in the 107 NPCs and 20 NNM by using qRT-PCR. We observed that miR-4465 expression was significantly decreased in the NPC tissues as compared with NNM (Fig. 4a). Next, we analyzed the effects of miR-4465 mimic on NPC cell proliferation, migration, and invasion. CCK-8, plate clone formation, and EdU incorporation assay showed that miR-4465 mimic dramatically inhibited in vitro NPC cell proliferation (Fig. 4b–e), and scratch wound healing and Transwell Matrigel invasion assay showed that miR-4465 mimic dramatically inhibited in vitro NPC cell migration (Fig. 4f) and invasion (Fig. 4g). Moreover, expression of exogenous EphA2 could antagonize the inhibitory effects of miR-4465 on NPC cell proliferation, migration, and invasion (Fig. 4b–g). The results indicate that miR-4465 functions as a tumor suppressor in NPC.

### HDAC7 promotes NPC cell proliferation, migration, and invasion by miR-4465 -EphA2 axis

**Fig. 5:**
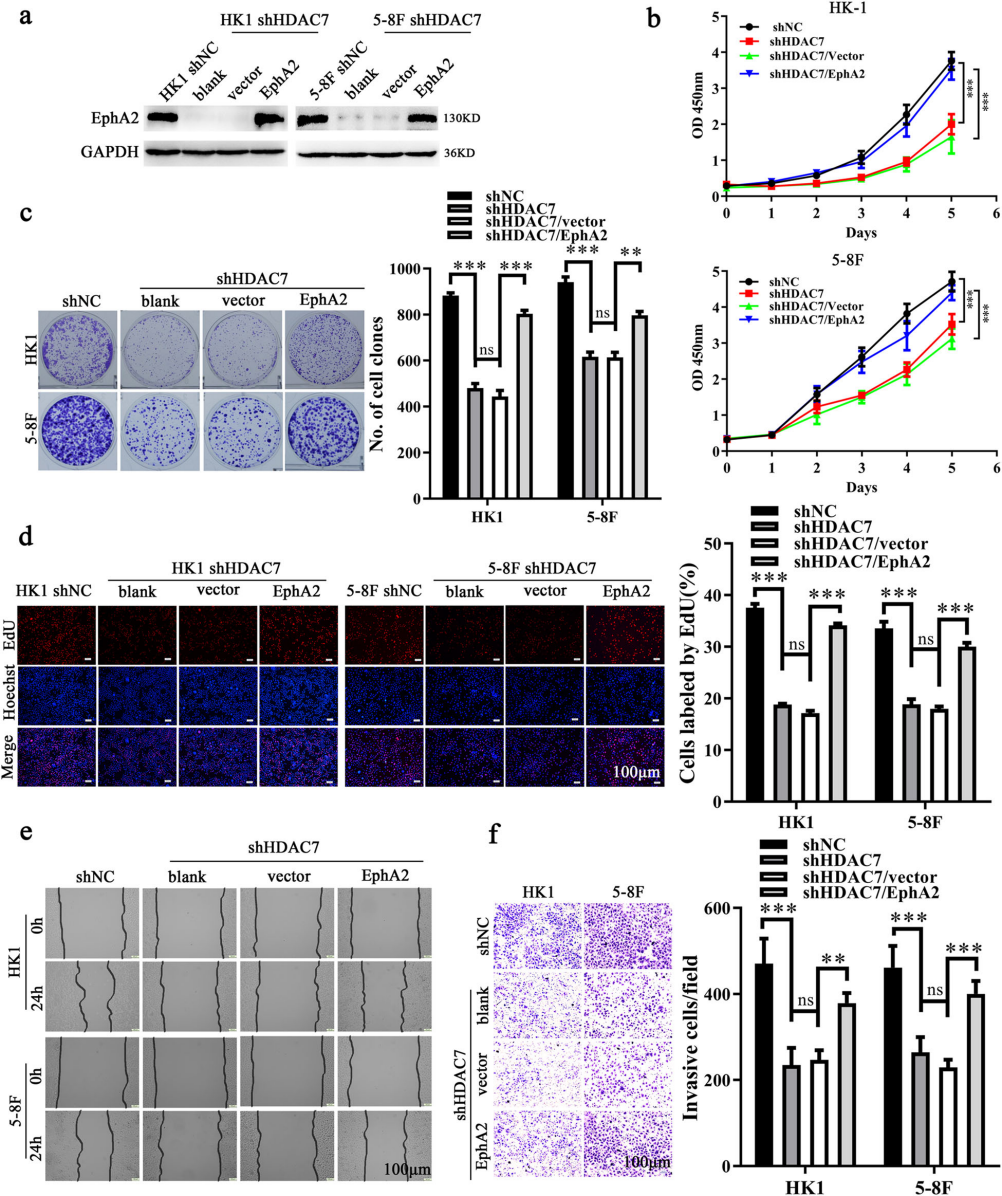
HDAC7 promotes NPC cell proliferation, migration, and invasion by upregulating EphA2. **a** Western blot showing the levels of EphA2 in the shHDAC7 HK1 and 5–8F cells transfected with EphA2 expression plasmid and their control cells. **b–f** Restoration of EphA2 expression antagonized the inhibitory effects of HDAC7 knockdown on NPC cell proliferation, and migration and invasion in vitro. CCK-8 (**b**), plate clone formation (**c**), and EdU incorporation (**d**) assay showing the proliferation of shHDAC7 HK1 and 5–8F cells transfected with EphA2 expression plasmid and their control cells. **e** Scratch wound healing showing the migration of shHDAC7 HK1 and 5–8F cells transfected with EphA2 expression plasmid and their control cells. **f** Transwell Matrigel invasion assay showing the invasion of shHDAC7 HK1 and 5–8F cells transfected with EphA2 expression plasmid and their control cells. Means, SDs, and statistical significance are denoted; **P < 0.01; ***P < 0.001; ns no significance.

First, we explored whether EphA2 mediates the NPC promotion of HDAC7. We transfected EphA2 expression plasmid into HK1 and 5–8F cells with HDAC7 knockdown, and then cell proliferation, migration, and invasion were detected. The results showed that restoration of EphA2 expression antagonized the inhibitory effects of HDAC7 knockdown on NPC cell proliferation (Fig. 5a–d), and migration and invasion (Fig. 5e, f). The results suggest that HDAC7 exerts its oncogenic roles by upregulating EphA2 in NPC cells.

**Fig. 6:**
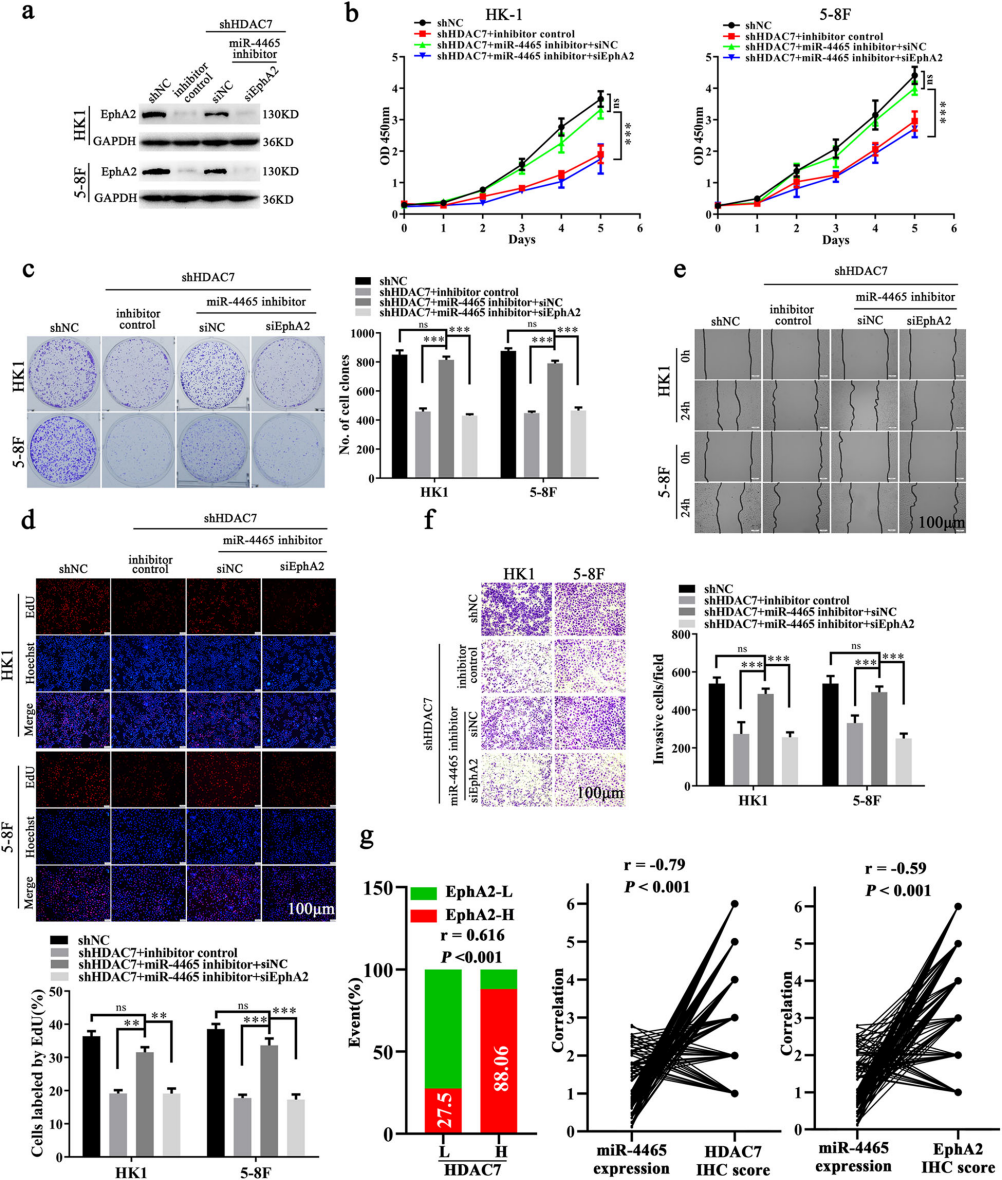
HDAC7 promotes NPC cell proliferation, migration, and invasion by miR-4465-EphA2 axis. **a** Western blot showing the levels of EphA2 in the shHDAC7 HK1 and 5–8F cells transfected with miR-4465 inhibitor or co-transfected with miR-4465 inhibitor and EphA2 siRNA (siEphA2), and their control cells. **b–f** The effects of miR-4465 inhibitor transfection or miR-4465 inhibitor and siEphA2 co-transfection on NPC cell proliferation, and migration and invasion. CCK-8 (**b**), plate clone formation (**c**), and EdU incorporation (**d**) assay showing the proliferation of shHDAC7 HK1 and 5–8F cells transfected with miR-4465 inhibitor or co-transfected with miR-4465 inhibitor and siEphA2, and their control cells. **e** Scratch wound healing assay showing the migration of shHDAC7 HK1 and 5–8F cells transfected with miR-4465 inhibitor or co-transfected with miR-4465 inhibitor and siEphA2, and their control cells. **f** Transwell Matrigel invasion assay showing the invasion of shHDAC7 HK1 and 5–8F cells transfected with miR-4465 inhibitor or co-transfected with miR-4465 inhibitor and siEphA2, and their control cells. **g** Correlation analyses of HDAC7, miR-4465, and EphA2 in 107 NPC based on their expression levels. (Left) Spearman correlation analysis showing the positive correlation between HDAC7 and EphA2. (Right) Spearman correlation analysis showing the negative correlation between miR-4465 and HDAC7, and miR-4465 and EphA2. Means, SDs, and statistical significance are denoted; **P < 0.01; ***P < 0.001; ns no significance.

Next, we explored whether miR-4465 mediates the NPC promotion of HDAC7 and EphA2. MiR-4465 inhibitor was transfected alone or co-transfected with EphA2 siRNA (siEphA2) into NPC cells with HDAC7 knockdown, and then cell proliferation, migration, and invasion were detected. The results showed that miR-4465 inhibitor antagonized the inhibitory effects of HDAC7 knockdown on NPC cell proliferation (Fig. 6a–d), and migration (Fig. 6e) and invasion (Fig. 6f). Moreover, the antagonized effects of miR-4465 inhibitor on HDAC7 knockdown cell proliferation, migration, and invasion were abolished when EphA2 was knocked down by siEphA2 (Fig. 6a–f). Collectively, these results demonstrate that HDAC7 promotes the oncogenicity of NPC cells by miR-4465-EphA2 axis.

### Levels of HDAC7, miR-4465, and EphA2 are correlated in human NPC biopsies

Since our data demonstrate that HDAC7 promotes the oncogenicity of NPC cells by miR-4465-EphA2 axis, we analyzed whether the levels of HDAC7, miR-4465, and EphA2 are correlated in human NPC biopsies. IHC showed that the expression of HDAC7 and EphA2 was significantly higher in NPCs than that in NNM (Fig. 1a). QRT-PCR observed that miR-4465 expression was significantly decreased in the NPC tissues as compared with NNM (Fig. 4a). Spearman correlation analysis showed that HDAC7 expression was positively associated with EphA2 level (*r* = 0.616, *P* < 0.001), whereas negatively associated with miR-4465 level (*r* = −0.79, *P* < 0.001), and miR-4465 level was negatively associated with EphA2 level (*r* = −0.59, *P* < 0.001) (Fig. 6g). Together, these results indicate that high HDAC7 expression appears to be associated with downregulation of miR-4465 and upregulation of EphA2 in the NPC tissues, and these misregulations might contribute to NPC development and progression.

## Discussion

HDAC7 is dysregulated in the many cancers^13–19^, is correlated with metastasis and poor prognosis, and is the main target of several HDACis^21–23^. Although several HDACis have exhibited anti-NPC effects^7–11^, the role and underlying mechanism of HDAC7 in NPC have not been explored. In the present study, we observed that HDAC7 is upregulated in NPCs, and correlated with NPC progression and poor prognosis, and promoted the in vitro proliferation, migration, and invasion and in vivo growth of NPC cells, indicating that HDAC7 functions as an oncogene.

Several studies indicate that oncogenic roles of HDAC7 are dependent on upregulation of Stat3 and c-Myc^13,14,39–41^. However, we did not observe the effects of HDAC7 knockdown on the expression of c-Myc and Stat3 in NPC cells (data not shown), suggesting that other factors mediate the function of HDAC7 in NPC. It has been reported that WW437, a novel HDACi, suppresses breast cancer progression by downregulating EphA2 via a HDAC2 and HDAC4 dependent manner^32^. Our recent study demonstrates that EphA2 and its phosphorylation at S897 are essential for NPC cell migration, metastasis, and stemness^33^. For this reason, we explored whether HDAC7 also upregulates EphA2 in the NPC cells, and found that HDAC7 absolutely upregulates EphA2. HDAC2 and HDAC4 transcriptionally upregulate EphA2 in the breast cancer cells^32^, however our results indicate that HDAC7 posttranscriptionally upregulates EphA2 in the NPC cells. We found that EphA2 is a direct target of miR-4465 in the NPC cells, and HDAC7 upregulates EphA2 by inhibiting miR-4465 expression. Moreover, our data further demonstrate that HDAC7 promotes the oncogenicity of NPC cells by inhibiting miR-4465 expression, and then upregulating EphA2, suggesting that HDAC7 is a potential therapeutic target for NPC. To our knowledge, it is the first time report that HDAC7 upregulates EphA2, and miR-4465 targets EphA2 expression in cancers.

HDAC7 is overexpressed in the many cancers^13–15,20,42^. Accumulated studies indicate that both transcriptional and posttranscriptional regulators are involved in upregulation of HDAC7. ZNF326, an oncogenic transcription factor, promotes HDAC7 transcription via binding to its promoter in glioma^18^. Several miRNAs, such as miR-34a^16^ and miR-489^43^, suppress HDAC7 expression by targeting HDAC7 mRNA degradation in the breast and colorectal cancer cells respectively. It has been reported that miR-34a, a negative regulator of HDAC7^16^, downregulates in the NPC and suppresses the malignant biological behaviors of NPC cells^44–46^. It is therefore plausible that miR-34a is involved in HDAC7 downregulation in NPC, which needs further experimental validation.

MiR-4465 is one member of miR-26 family, however the role of miR-4465 in cancer was rarely reported^37,38^. Therefore we explored whether miR-4465 is involved in NPC, and found that miR-4465 expression was significantly decreased in the NPC tissues as compared with NNM, and miR-4465 mimic dramatically inhibited NPC cell proliferation, migration, and invasion. Our data indicate that miR-4465 functions as a tumor suppressor in NPC, providing additional evidences for the tumor suppressing function of miR-4465. Our results showed that EphA2 also was a predictive target of miR-26b-5p, and miR-26b-5p mimic remarkably decreased EphA2 protein levels in NPC cells, supporting that EphA2 is a direct target of miR-26b-5p in the cancers^47,48^.

In summary, our data reveal that HDAC7 functions as an oncogene in NPC, and promotes the oncogenicity of NPC cells by inhibiting miR-4465 expression, and subsequently upregulating EphA2, and miR-4465 functions as a tumor suppressor in NPC. Our findings indicate that HDAC7 is a potential therapeutic target in NPC.

## Materials and methods

### Patients and tissue samples

The one hundred and seven formalin-fixed and paraffin-embedded archival NPC tissue specimens, and twenty NNM between Jan 2010 and Dec 2012 were obtained from the Xiangya Hospital of Central South University at the time of diagnosis before any therapy. In addition, four paired fresh NPC and NNM biopsies were also collected. On the basis of the 1978 WHO classification^33,49^, all tumors were histopathologically diagnosed as poorly differentiated squamous cell carcinomas (WHO type III). The clinical stage of the patients was classified and reclassified according to the AJCC criteria as described in the seventh edition of the AJCC cancer staging manual^33,49^. All the patients received radiochemotherapy according to a uniform guideline, and data on clinicopathological features and prognoses of the patients were collected and analyzed retrospectively. The follow-up period was 9–71(50 ± 18.3) months. OS was defined as the time from the initiation of primary therapy to the date of cancer-related death or when censured at the latest date if patients were still alive. DFS was calculated as the time from the completion of primary radiotherapy to the date of pathological diagnosis or clinical evidence of local failure and/or distant metastasis. The clinicopathological features of the patients used in the present study are shown in Supplementary Table S3.

### Cell lines and plasmids

Human NPC cell line HK1 was kindly gifted by Dr. Tao of the Chinese University of Hong Kong, and human NPC cell lines 5–8F, 6–10B, CNE1 and CNE2 and NP69, an immortalized normal human nasopharynx epithelial cell line, have been described previously by us^33,49,50^. NPC cells were cultured with RPMI-1640(BI, Israel) medium supplemented with 10% fetal bovine serum (BI) at 37 °C in 5% CO_2_. NP69 cells were cultured with Defined Keratinocyte-SFM medium (Thermo Fischer Scientific) at 37 °C in 5% CO_2_. The cell lines were authenticated by short tandem repeat profiling prior to use, and were routinely tested negative for mycoplasma contamination using 4,6-diamidino-2-phenylindole staining.

Lentiviral interfering plasmid GV248-shHDAC7 and scramble nontarget shRNA control plasmid GV248-shNC, and a dual-luciferase reporter plasmid expressing wild-type EphA2 3′-UTR or mutant EphA2 3′-UTR in the predicted miR-4465 binding site were established by Genechem Inc. (Shanghai, China), and confirmed by sequencing. The core target sequence of shHDAC7 was 5′-GGCUGGAAACAGAAACCCA-3′. pENTER-HDAC7 expression plasmid and control plasmid were purchased from Vigene bioscience Inc. EphA2 expression plasmid and control plasmid have been described previously by us^33^.

### Establishment of NPC cell lines with HDAC7 knockdown

HK1 and 5–8F cells were infected with lentiviral GV248-shHDAC7 and GV248-shNC particles respectively, and were selected using puromycin for 2 weeks, then HK1 and 5–8F cell lines with the stable knockdown of HDAC7, and their control cell lines were obtained.

### Transient transfection

MiR-4465, miR-26b-5p and miR-1297 mimic, miR-4465 and miR-26b-5p inhibitor and their respective negative control, and EphA2 and HDAC7 3′UTR (untranslated region) siRNA and their respective control siRNA, which were purchased from Ribobio Inc. (Guangzhou, China), were transfected into the indicated cells using riboFect™ CP Transfection Kit (Ribobio Inc.) according to the manufacturer’s instructions respectively. The target sequence of EphA2 and HDAC7 siRNA was 5′-CAGCCTTCGGACAGACATA-3′, and 5′-CAAGTAGTTGGAACCAGAGAA-3′, respectively. EphA2 and HDAC7 expression plasmid and their control plasmid were transfected into the indicated cells by using Lipofectamine^TM^ 2000 (Thermo Fischer Scientific) according to the manufacturer’s instruction respectively.

### Western blot

Western blot was performed as described previously by us^33,49^. Briefly, proteins were exacted from cells or tissues using RIPA lysis buffer. An equal amount of protein in each sample was subjected to SDS-PAGE separation, followed by blotting onto a PVDF membrane. After blocking, blots were incubated with rabbit anti-HDAC7 antibody (A7258, ABclonal; 1:500 dilution), mouse anti-EphA2 antibody (sc-398832, Santa Cruz; 1:100 dilution) overnight at 4 °C, followed by incubation with HRP-conjugated secondary antibody (Abcam, 1:5000 dilution) for 1 h at room temperature. The signal was visualized with chemiluminescence detection reagent (Millipore).

### RNA quantification

QRT-PCR was performed as described previously by us^49,51^. Briefly, total RNA was extracted from the indicated NPC cells with Trizol reagent (Thermo Fischer Scientific), or from the paraffin-embedded tissues of 107 NPCs and 20 NNMs with RecoverAll^TM^ total nucleic acid isolation kit (Ambion) according to the manufacturer’s instructions. For miRNA qRT-PCR, miDETECT A Track^TM^ miRNA qRT-PCR Starter Kit (RiboBio Inc.) was used to reversely transcribe the total RNA to cDNA and amplify the RT product according to the manufacturer’s instructions. For EphA2 mRNA qRT-PCR, a RT kit and Oligo dT primer (Promega) was used to reversely transcribe the total RNA to cDNA, and the RT products were amplified by real-time PCR using QuantiFast SYBR green PCR kit (Qiagen) according to the manufacturer’s instructions. The products were quantitated using 2^−*DDCt*^ method against 5S or GAPDH for normalization. The primer sequences were synthesized by RiboBio Inc. and summarized in the Supplementary Table S4. All assays were performed three times in triplicate.

### Luciferase activity assay

Dual-luciferase reporter system assay was performed as described previously by us^49^. Briefly, a dual-luciferase reporter plasmid with wild-type EphA2 3′-UTR or mutant EphA2 3′-UTR was co-transfected with miR-4465 mimic or mimic control into HEK293 cells using Lipofectamine 2000 respectively. Cells were harvested 48 h after transfection, both firefly luciferase and renilla luciferase activities were measured using the dual-luciferase reporter assay system (Promega) according to the manufacturer’s instructions, and luciferase activity was estimated using a luminometer (Promega). The assay was performed three times in triplicate.

### Cell Counting Kit-8 (CCK-8) assay

Cell proliferation was measured using a CCK-8 kit as described previously by us^49,52^. The assay was performed three times in triplicate.

### Plate clone formation assay

Plate colony formation assay was performed to detect cell proliferation described previously by us^49,52^. The assay was performed three times in triplicate.

### 5-ethynyl-2′-deoxyuridine (EdU) incorporation assay

EdU incorporation assay was performed to detect cell proliferation as described previously by us^49,52^. The assay was performed three times in triplicate.

### Scratch wound healing and Transwell Matrigel invasion assay

Scratch wound healing and matrigel invasion assay was performed to detect cell migration and invasion as described previously by us^33,53^. All assays were performed three times in triplicate.

### Tumor formation assay in nude mice

Nude female Balb/c mice that were 4 weeks old were obtained from the Laboratory Animal Center of Central South University (Changsha, China) and were maintained under specific pathogen-free conditions. For tumor formation experiment, 5 × 10^6^ cells resuspended in 200 μl of medium without serum were subcutaneously injected into the flanks of mice (*n* = 5 mice each). The mice were monitored daily for palpable tumor formation, and tumor volume (in mm^3^) was measured by a vernier caliper every 4 days and calculated by using the modified ellipse formula (volume = length × width^2^/2). After 20 days, the mice were killed by cervical dislocation, and their tumors were excised, weighted, and embedded in paraffin.

### Immunohistochemistry and staining evaluation

IHC were performed on the formalin-fixed and paraffin-embedded tissue sections as described previously by us^33,49,53^. Briefly, tissue sections were incubated with anti-HDAC7 antibody (D160484–0200, BBI Life Sciences; 1:50 dilution) or anti-EphA2 antibody (6997, CST; 1:200 dilution) overnight at 4 °C, and then incubated with biotinylated secondary antibody followed by avidin-biotin peroxidase complex (DAKO) at room temperature for 30 min. Finally, tissue sections were incubated with 3′, 3′-diaminobenzidine (Sigma-Aldrich) and counterstained with hematoxylin. In negative controls, primary antibodies were replaced with a normal mouse or rabbit IgG. Known immunostaining-positive slides were used as positive controls.

Immunohistochemical staining was assessed and scored by two independent pathologists who were blinded to the clinicopathological data. Discrepancies were resolved by consensus. Staining intensity was categorized: absent staining as 0, weak as 1, moderate as 2, and strong as 3. The percentage of stained cells (examined in at least 500 cells) was categorized as no staining = 0, <30% of stained cells = 1, 30–60% = 2, and >60% = 3. The staining score (ranging from 0 to 6) for each tissue was calculated by adding the area score and the intensity score. A combined staining score of ≤3 was considered to be low expression, and >3 was considered to be high expression.

### Statistical analysis

Statistical analysis was performed using IBM SPSS statistical software package 22. Data are presented as means ± SD. Qualitative variables were compared by the Student’s *t* test or Chi-square test. Kaplan–Meier survival analysis was used to compare NPC patient survival by the log-rank test. Cox proportional hazards regression analyses were used to analyze the effect of clinical variables on patient survival. The Spearman rank correlation coefficient was used to determine the correlation between the two variables. All statistical tests were two-sided, and the *p* values <0.05 were considered statistically significant.

### Ethics statement

The use of human tissues was approved by the Ethics Committee of Xiangya Hospital, Central South University. As only archived tumor specimens were included in this study, the ethics committee waived the need for consent and patient records/information were analyzed anonymously. All animal experimental procedures were performed in accordance with the Guide for the Care and Use of Laboratory Animals of Xiangya Hospital, Central South University, with the approval of the Institutional Animal Ethics Committee.

## Supplementary information

Supplementary Tables

Supplementary Figure Legends

Supplementary Figure S1

Supplementary Figure S2

Supplementary Figure S3

Supplementary Figure S4

Supplementary Figure S5
