# Supplementary material for: HDAC7 promotes the oncogenicity of nasopharyngeal carcinoma cells by miR-4465-EphA2 signaling axis
Source: Cell Death Dis. 2020 May 6;11(5):322. doi: 10.1038/s41419-020-2521-1 (PMC7203158; doi:10.1038/s41419-020-2521-1)
Supplement: Supplementary file 1 — Supplementary Tables [file 41419_2020_2521_MOESM1_ESM.docx]

**Supplementary Table S1. Correlation between HDAC7 expression and clinicopathological characteristics in NPCs (n=107, χ2 test)**

| **Variables** | **n** | **Expression level** | | ***P*** |
| --- | --- | --- | --- | --- |
|  |  | **Low (0-3)** | **High (4-6)** |  |
| **Age(ys)** |  |  |  |  |
| ≥48 | 66 | 24 | 42 | 0.470 |
| <48 | 41 | 16 | 25 |  |
| **Gender** |  |  |  |  |
| Male | 58 | 22 | 36 | 0.530 |
| Female | 49 | 18 | 31 |  |
| **Primary tumor (T) stage** |  |  |  |  |
| T1-2 | 30 | 16 | 14 | 0.029 |
| T3-4 | 77 | 24 | 53 |  |
| **Lymph node(N) metastasis** |  |  |  |  |
| N0 | 46 | 22 | 24 | 0.041 |
| N1-3 | 61 | 18 | 43 |  |
| **Distant metastasis (M)** |  |  |  |  |
| M0 | 80 | 37 | 43 | 0.001 |
| M1 | 27 | 3 | 24 |  |
| **Clinical TNM stage** |  |  |  |  |
| I-II | 53 | 29 | 24 | <0.001 |
| III-IV | 54 | 11 | 43 |  |

**Supplementary Table S2. Univariate and multivariate analyses of selected prognostic factors for** **overall free survival and disease free survival using Cox proportional hazards** **regression model (n=107)**

| **Variable** | **Overall survival(OS)** | | | | |  | **Disease free survival(DFS)** | | | | |
| --- | --- | --- | --- | --- | --- | --- | --- | --- | --- | --- | --- |
|  | **Univariate analysis** | |  | **Multivariate analysis** | |  | **Univariate analysis** | |  | **Multivariate analysis** | |
|  | ***P*** | **HR (95% CI)** |  | ***P*** | **HR(95% CI)** |  | ***P*** | **HR (95% CI)** |  | ***P*** | **HR(95% CI)** |
| **Age(ys)** |  |  |  |  |  |  |  |  |  |  |  |
| ≥48 *vs* *<*48 | 0.490 | 1.222（0.692-2.158） |  | 0.530 | 1.849（0.991-3.449） |  | 0.563 | 1.175（0.679-2.033） |  | 0.213 | 1.452（0.807-2.615） |
| **Gender** |  |  |  |  |  |  |  |  |  |  |  |
| Male *vs.* Female | 0.774 | 1.083（0.630-1.860） |  | 0.114 | 1.685（0.883-3.217） |  | 0.364 | 1.275（0.754-2.158） |  | 0.848 | 1.078（0.501-2.318） |
| **Primary tumour (T) stage** |  |  |  |  |  |  |  |  |  |  |  |
| T3-4 *vs.*T1-2 | 0.001 | 3.152（2.046-8.969） |  | 0.004 | 2.175（0.759-6.231） |  | <0.001 | 5.847（2.326-9.695） |  | 0.049 | 2.860（1.003-8.159） |
| **Lymph node (N) metastasis** |  |  |  |  |  |  |  |  |  |  |  |
| N1-3 *vs.*N0 | 0.001 | 2.716（1.472-5.013） |  | 0.030 | 0.380（0.159-0.913） |  | <0.001 | 2.985（1.642-5.427） |  | 0.002 | 0.973（0.232-1.353） |
| **Distant metastasis (M)** |  |  |  |  |  |  |  |  |  |  |  |
| M1 *vs.* M0 | <0.001 | 2.694（1.482-4.825） |  | 0.005 | 1.473（0.629-3.135） |  | <0.001 | 5.032（4.713-9.302） |  | 0.037 | 2.321（1.051-5.124） |
| **Clinical TNM stage** |  |  |  |  |  |  |  |  |  |  |  |
| III-IV *vs.* I-II | <0.001 | 2.920（1.093-4.415） |  | 0.001 | 2.964（1.220-5.126） |  | <0.001 | 5.144（4.090-9.620） |  | 0.001 | 3.968（2.520-5.124） |
| **HDAC7 level** |  |  |  |  |  |  |  |  |  |  |  |
| High *vs.* Low | <0.001 | 4.023（2.014-8.035） |  | 0.004 | 3.108（1.428-6.766） |  | <0.001 | 3.937（2.086-7.430） |  | 0.001 | 3.272（1.588-6.739） |

**Supplementary Table S3. The clinicopathological parameters of 107 patients with NPC**

| **Variable** | **No. of patients** | **%** |
| --- | --- | --- |
| **Age(ys)**  ≥48 | 66 | 61.7 |
| <48 | 41 | 38.3 |
| **Gender** |  |  |
| Male | 58 | 54.2 |
| Female | 49 | 45.8 |
| **Primary tumor(T) stage** |  |  |
| T1-2 | 30 | 28.0 |
| T3-4 | 77 | 72.0 |
| **Lymph node(N) metastasis** |  |  |
| N0 | 46 | 43.0 |
| N1-3 | 61 | 57.0 |
| **Distant metastasis(M)** |  |  |
| M0 | 80 | 74.8 |
| M1 | 27 | 25.2 |
| **Clinical TNM stage** |  |  |
| I-II | 53 | 49.5 |
| III-IV | 54 | 50.5 |

**Supplementary Table S4. qRT-PCR primers for amplification of microRNA and mRNA synthesized by Ribobio Inc.**

| **No.** | **Name** | **RT primer** | | **Primer sequence** |
| --- | --- | --- | --- | --- |
| 1 | miR-4465 | | Cat.#,miRA1000178 | F: Cat.#, miRA1000178 |
|  |  |  |  | R:Cat.#,miRAU0005 |
| 2 | miR-26b-5p | | Cat.#,miRA0000083 | F: Cat.#, miRA0000083 |
|  |  |  |  | R: Cat.#,miRAU0005 |
| 3 | 5S | | Cat.#, miRAN0001 | F: Cat.#, miRAN0001 |
|  |  |  |  | R: Cat.#,miRAU0005 |
| 4 | EphA2 | | Oligo dT | F:5’-TGGCTCACACACCCGTATG-3’ |
|  |  |  |  | R:5’-GTCGCCAGACATCACGTTG-3’ |
| 5 | EphA2 Intron 1 | | Oligo dT | F:5’-GAGCGTGGAGATAGGTTCGG-3’ |
|  |  |  |  | R:5’-TCGTTCTGAGTCACCCTTGC-3’ |
| 6 | EphA2 Intron 5 | | Oligo dT | F:5’-CTGTGGAGGATGTGGCTCC-3’ |
|  |  |  |  | R:5’-GTGGGGGAAGTGGGTAAGAAG-3’ |
| 7 | EphA2 Intron 16 | | Oligo dT | F:5’-TCCTCGCCAATTCCTTTCCC-3’ |
|  |  |  |  | R:5’-CAGGCCCTTTCACAACAACG-3’ |
| 8 | GAPDH | | Oligo dT | F:5’-TGACTTCAACAGCGACACCCA-3’ |
|  |  |  |  | R:5’-CACCCTGTTGCTGTAGCCAAA-3’ |
