# Supplementary material for: HDAC7 promotes the oncogenicity of nasopharyngeal carcinoma cells by miR-4465-EphA2 signaling axis
Source: Cell Death Dis. 2020 May 6;11(5):322. doi: 10.1038/s41419-020-2521-1 (PMC7203158; doi:10.1038/s41419-020-2521-1)
Supplement: Supplementary file 2 — Supplementary Figure Legends [file 41419_2020_2521_MOESM2_ESM.docx]

**Supplementary Figure S1**. Expression of exogenous HDAC7 antagonizes the inhibitory effects of HDAC7 knockdown on NPC cells. (**a**) Western blot showing the levels of HDAC7 and EphA2 in the HK1 and 5-8F NPC cells cotransfected with HDAC7 3’ UTR siRNA and HDAC7 expression plasmid, and their control cells. CCK-8 (**b**) and EdU incorporation (**c**) assay showing the proliferation of HK1 and 5-8F NPC cells cotransfected with HDAC7 3’ UTR siRNA and HDAC7 expression plasmid, and their control cells. Scratch wound healing (**d**) and Transwell Matrigel invasion (**e**) assay showing the migration and invasion of HK1 and 5-8F NPC cells cotransfected with HDAC7 3’ UTR siRNA and HDAC7 expression plasmid, and their control cells. Means, SDs, and statistical significance are denoted; *** *P* <0.001; ns, no significance.

**Supplementary Figure S2. The effects of miR-26b-5p and miR-1297 mimic on EphA2 protein expression in the NPC cells.** Western blot showing the levels of EphA2 in the HK1 and 5-8F cells transfected with 100nM miR-26b-5p, miR-1297 mimic or mimic control.

**Supplementary Figure S3.** **HDAC7 knockdown does not affect miR-26b-5p expression in the NPC cells.** QRT-PCR showing the levels of miR-26b-5p in the HK1 and 5-8F cells with shHDAC7 and control cells. Means, SDs, and statistical significance are denoted; ns, no significance.

**Supplementary Figure S4.** **MiR-4465 is upregulating in the xenografts generated by NPC cells with HDAC7 knockdown.** QRT-PCR showing the expression levels of miR-4465 in the xenografts generated by HK1 and 5-8F cells with shHDAC7 and control cells. Means, SDs, and statistical significance are denoted; ***, *P* <0.001.

**Supplementary Figure S5. MiR-26b-5p inhibitor dose not restore the levels of EphA2 protein in NPC cells with HDAC7 knockdown.** Western blot showing the levels of EphA2 in the shHDAC7 HK1 and 5-8F cells transfected with 200nM miR-26b-5p inhibitor or inhibitor control.
