# Supplementary figures and images for: HDAC7 promotes the oncogenicity of nasopharyngeal carcinoma cells by miR-4465-EphA2 signaling axis
Source: Cell Death Dis. 2020 May 6;11(5):322. doi: 10.1038/s41419-020-2521-1 (PMC7203158; doi:10.1038/s41419-020-2521-1)

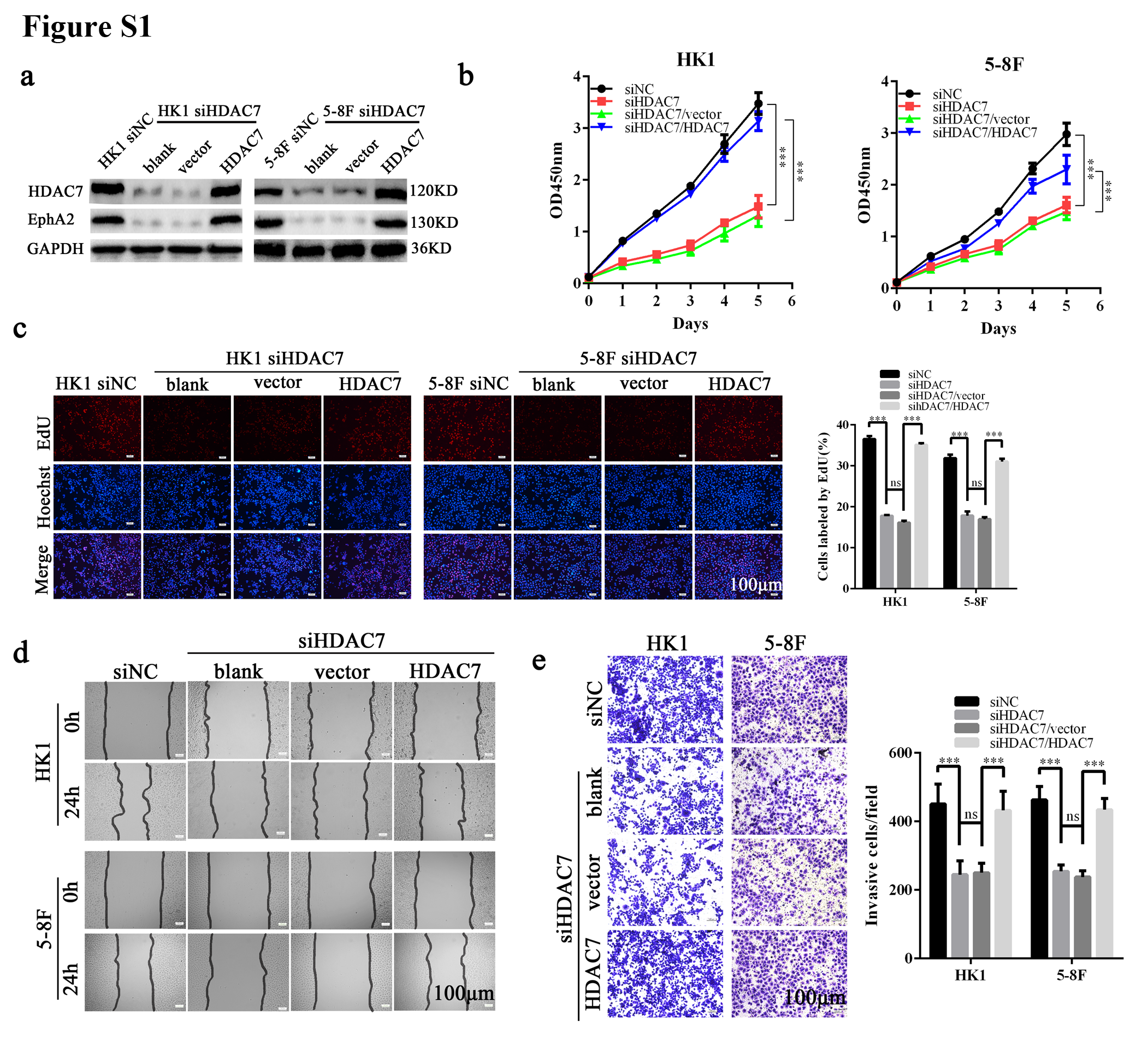

Supplement: Supplementary file 3 — Supplementary Figure S1 [file 41419_2020_2521_MOESM3_ESM.tif]

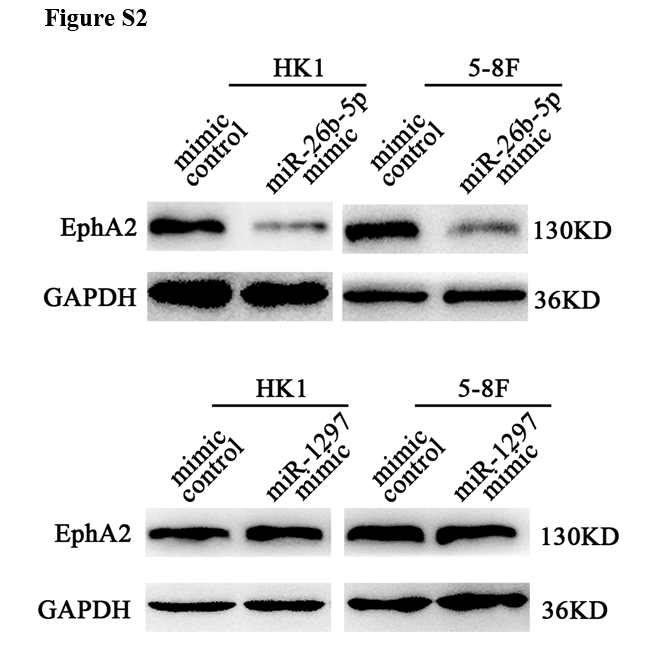

Supplement: Supplementary file 4 — Supplementary Figure S2 [file 41419_2020_2521_MOESM4_ESM.tif]

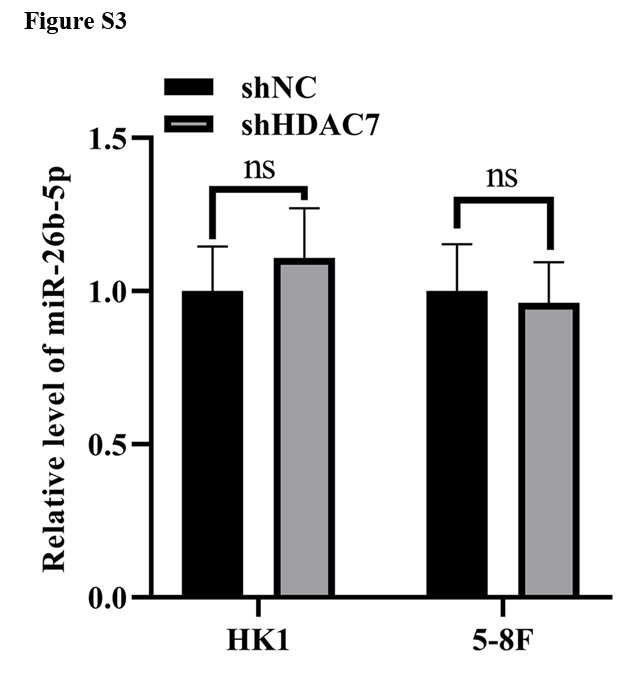

Supplement: Supplementary file 5 — Supplementary Figure S3 [file 41419_2020_2521_MOESM5_ESM.tif]

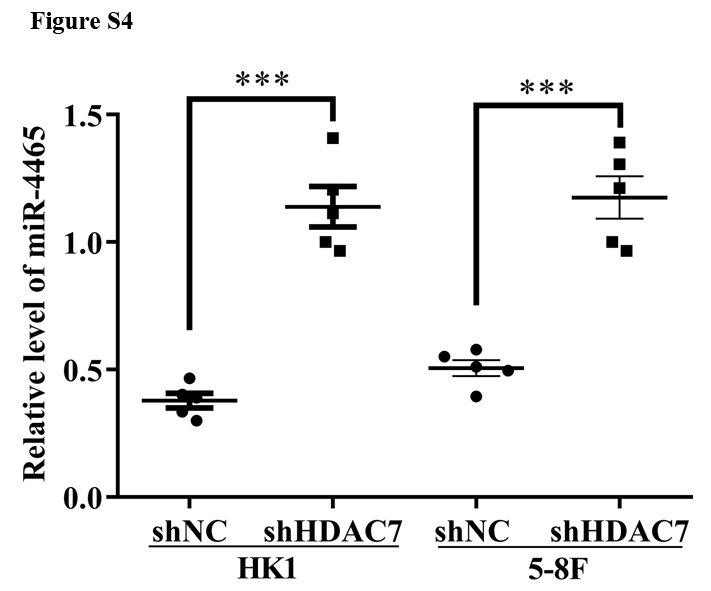

Supplement: Supplementary file 6 — Supplementary Figure S4 [file 41419_2020_2521_MOESM6_ESM.tif]

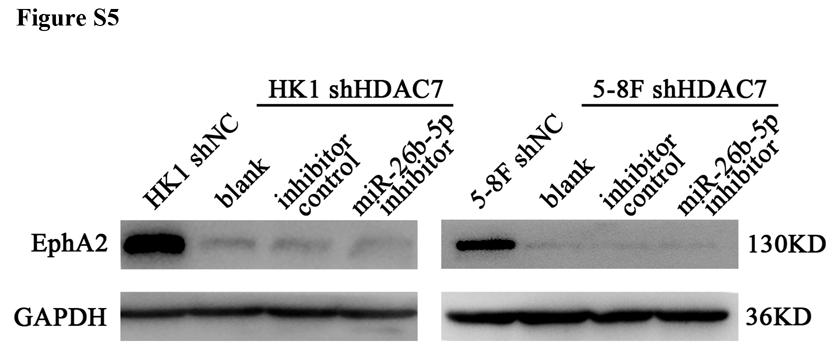

Supplement: Supplementary file 7 — Supplementary Figure S5 [file 41419_2020_2521_MOESM7_ESM.tif]
